# Circulation and Spillover of pdmH1N1 Influenza A Virus at an Educational Swine Farm in Chile, 2019–2023

**DOI:** 10.3390/v17050635

**Published:** 2025-04-28

**Authors:** Soledad Ruiz, Constanza Díaz-Gavidia, María Antonieta González, Pablo Galdames, Cristóbal Oyarzún, Cecilia Baumberger, Camila Rojas, Christopher Hamilton-West, Bridgett Sharp, Shaoyuan Tan, Stacey Schultz-Cherry, Pedro Jimenez-Bluhm

**Affiliations:** 1 Escuela de Medicina Veterinaria, Facultad de Recursos Naturales y Medicina Veterinaria, Universidad Santo Tomás, Santiago 8370003, Chile; soleruizp@gmail.com; 2 Escuela de Medicina Veterinaria, Facultad de Medicina, Facultad de Ciencias Biológicas, y Facultad de Agronomía y Sistemas Naturales, Pontificia Universidad Católica de Chile, Santiago 7820436, Chile; diaz.constanzag@gmail.com (C.D.-G.);; 3 Departamento de Medicina Preventiva Animal, Facultad de Ciencias Veterinarias y Pecuarias, Universidad de Chile, Santiago 8820000, Chilechristopher.hamilton@veterinaria.uchile.cl (C.H.-W.); 4 Host-Microbe Interactions, St. Jude Children’s Research Hospital, Memphis, TN 38105, USAstacey.schultz-cherry@stjude.org (S.S.-C.)

**Keywords:** zoonotic transmission, influenza A virus, educational farms, swine surveillance, reverse zoonosis

## Abstract

Educational farms provide students with hands-on experience in agricultural and animal practices. However, the close contact between humans and farm animals creates a significant interface for zoonotic disease transmission, yet research on infectious diseases in such settings remains limited. This study investigates the ongoing spillovers of human-origin influenza A virus (IAV) into swine at an educational farm in central Chile, describing IAV prevalence, outbreak dynamics, and the genomic characterization of detected strains. The Menesianos educational farm, located in Melipilla, central Chile, houses approximately 40 swine alongside other domestic animals, such as horses and cows. As part of an active IAV surveillance project, monthly nasal swab samples were collected from pigs between June 2019 and September 2023 for IAV detection via RT-qPCR targeting the M gene, with positive samples subsequently sequenced. During the study period, monthly IAV prevalence ranged from 0% to 52.5%, with a notable outbreak detected between May and June 2023. The outbreak lasted 5 weeks, peaking at 52.5% prevalence during week 3. Nine IAV strains were isolated over the study period, eight of which were obtained during weeks 2 and 3 of the outbreak. Phylogenetic analysis revealed that all strains were closely related to the pandemic H1N1 2009 influenza virus, with the closest related strains being those circulating in humans in Chile during the same years. These findings highlight the importance of conducting regular IAV surveillance on educational farms, where close interactions between animals and individuals—particularly children and young people—can facilitate viral spillovers and potential reverse zoonosis events.

## 1. Introduction

Influenza A virus (IAV) is an endemic pathogen in swine populations worldwide, causing mild to severe upper respiratory tract illness [1]. The H1N1, H1N2, and H3N2 viruses are the most common IAV subtypes circulating in pigs in most countries of the world, although the genetic characteristics of the viruses vary considerably across different regions and several lineages have been reported [1,2]. The major lineages include swine-adapted viruses of North America, Europe, and Asia and human seasonal viruses that have spilled over into swine and become established, including the pandemic H1N1 virus (H1N1pdm09) [3]. Swine are considered critical hosts for IAV because they are recognized as important “mixing vessels” in which IAVs from multiple host species can reassort, representing a risk for the emergence of a novel influenza A virus with pandemic potential [4]. Examples of this include the emergence of the H1N1pdm09 strain, resulting from reassortment between avian and swine IAV strains in swine, and the variant influenza A(H3N2)v virus, which has caused several human infections in the United States [5,6,7].

In Chile, the information available about IAV circulation in swine is limited; however, studies reveal a high IAV prevalence and seroprevalence in both industrial farms and backyard production systems (BPS) [8,9,10,11,12]. Phylogenetic analysis revealed the circulation of three predominant subtypes in commercial farms: two novel and distinct lineages of the swine H1N2 subtype, a swine H3N2, and the A(H1N1) pdm09-like subtype [12]. Of these lineages, the H1N2 and the H3N2 viruses are related to human viruses from the late 1980s to mid-1990s but contain the internal genes of the A(H1N1)pdm09 pandemic strain [8].

In BPS, studies conducted in central Chile have shown that there is a wide circulation of IAV in pigs raised in these productive systems [9,10,11]. A one-year sampling effort in 113 BPS in the Libertador Bernardo O’Higgins (LGB O’Higgins) region showed that 2.4% of swine were positive for IAV by enzyme-linked immunosorbent assay (ELISA), indicative of previous exposure of farm animals to IAV [10]. Another study that included BPS from the Valparaiso, Metropolitan, and LGB O’Higgins regions determined a prevalence of IAV in swine of 6.3% measured by M-gene-specific RT-qPCR [9]. Furthermore, an H1N2 virus from swine in a BPS was isolated [11]. Genetically, this H1N2 virus had both HA and NA genes most like human viruses circulating in the 1980s and early 1990s, whereas the internal genes were similar to 2009 H1N1 pandemic viruses, having 100% nucleotide homology to strains circulating in commercial swine at the time [8]. The virus replicated efficiently in vitro and in vivo, was transmitted in ferrets by respiratory droplets, and might pose a risk to immunologically naïve persons born after 1990 [12].

Zoonotic infections and localized outbreaks of IAV in people have been widely documented and areas commonly associated with swine–human interfaces, such as agricultural fairs, live animal markets, and swine farms [13,14,15,16,17,18,19]. In fact, in the United States, the largest outbreaks of swine-to-human IAV transmission have occurred at agricultural fairs [14,17,20,21]. In the United States, a small portion of the swine population is raised in small farm settings and exhibited at agricultural fairs as part of educational projects to expand youth knowledge about agricultural practices [22]. Close contact between swine and humans at agricultural fairs provides an opportunity for bidirectional transmission of influenza A viruses between pigs and humans [14,17,22,23,24]. Since 2005, a total of 437 people have been infected by variant H3N2 (H3N2v) viruses originating in swine at agricultural fairs held in the United States [25]. This precedent indicates that swine exhibitions pose a significant risk for swine-lineage influenza A virus (IAV) amplification and zoonotic transmission. However, other interfaces that also have pigs on exhibition, such as educational farms, remain poorly studied.

An educational farm is a farm that regularly welcomes students (children and young people), with the aim of teaching them about activities related to specific agricultural and animal practices [26]. In many countries, educational farms are part of educational tourism in agriculture, which has been gaining popularity in recent years as an emerging potential market segment of rural tourism [27]. On these farms, guests take a tour around the site where they learn about a working farm and about the daily lives of farmers and their animals [26]. The close contact between humans and farm animals provides an important interface for the transmission of zoonotic diseases, such as IAV. However, research on infectious disease transmission between different hosts in such settings remains limited.

This study describes the ongoing spillovers of human-origin IAV into swine at an educational farm in central Chile, IAV prevalence, and the isolation and genomic characterization of IAV strains that affected the farm.

## 2. Materials and Methods

### 2.1. Study Area and Nasal Swab Sampling

The Menesianos educational farm, located in Melipilla, central Chile, houses approximately 40 pigs alongside other domestic animals, such as horses and cows. As part of an ongoing active IAV surveillance project, swine on this farm were sampled monthly from June 2019 to September 2023. This monitoring enabled the detection of an IAV outbreak between May and June 2023, during which sampling frequency increased to once a week.

On each sampling occasion, nasal swab samples were collected using sterile swabs (Copan^®^, Brescia, Italy) and stored in tubes containing 1 mL of universal transport medium (UTM, Copan^®^, Italy). Samples were maintained at 4 °C until arrival at the Infectious Diseases laboratory of the School of Veterinary Medicine of the Pontifical Catholic University of Chile. Samples were stored at −80 °C until processing. In addition, clinical signs and the approximate age of the sampled animals were recorded.

### 2.2. RNA Extraction and Molecular Diagnosis

RNA extraction and quantitative reverse transcription PCR were performed as described [28]. Briefly, viral RNA extraction was performed on 50 μL of a swab sample using the Ambion MagMax-96 AI/ND viral isolation kit (Life Technologies Corporation, Grand Island, NY, USA). Samples were screened for influenza A virus using RT-qPCR (Stratagene mx3000p, Santa Clara, CA, USA) with the TaqMan Fast Virus 1-Step Master Mix (Applied Biosystems, Foster City, CA, USA) and primers/probe specific to the influenza M gene [29]. Samples with a fluorescence cycle threshold value (Ct) ≤ 38 were considered positive [30]. For swab samples that tested positive by RT-qPCR with a Ct ≤ 35, a multi-segment amplification for NGS was performed, and then purification of DNA amplicons was performed to send for sequencing. Sample sequencing was carried out at the St. Jude Children’s Hospital Hartwell Center on the Illumina platform on a MiSeq sequencer (Illumina, San Diego, CA, USA). Sequences were de novo assembled and edited using the SPADes package, and the nucleotide sequences were deposited into the NCBI database and/or uploaded to the GISAID database (Supplementary Table S1) [31].

### 2.3. Genetic and Phylogenetic Analyses

Phylogenetic analysis included sequences of Influenza from swine and human hosts downloaded from the National Center for Biotechnology Information (NCBI) Influenza Virus Database or the Global Initiative on Sharing All Influenza Data (GISAID) database. All accession numbers of the viruses used to construct the phylogenetic trees in this study are listed in Supplementary Table S2. Sequences were then aligned with MUSCLE v5.3 [32] with manual correction and curation in BioEdit v7.7.1. Maximum likelihood trees were generated in the RAxML (v8.2.13) program [33] or the eight gene segments with the general time reversible (GTR) substitution model and gamma distribution of rate heterogeneity. To evaluate the reliability of tree topologies, bootstrapping analyses were conducted using 1000 replicates. All trees were rooted at the midpoint and visualized in iTol v7 [34]. Nucleotide and aminoacidic identity and similarity between strains were determined with BioEdit v7.7.1.

Additional clade assignment and amino acid substitutions that could potentially increase the susceptibility of pigs to human influenza viruses were evaluated using the FluSurver platform (https://flusurver.bii.a-star.edu.sg/, accessed on 8 April 2024), following default settings.

## 3. Results

**Figure 1:**
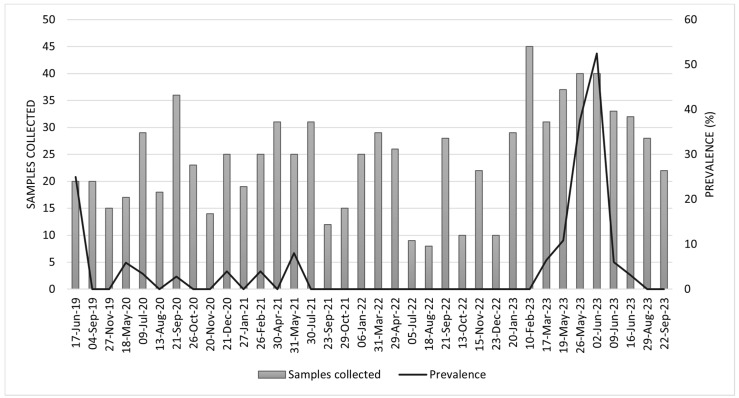
Monthly distribution of samples collected and prevalence rates from June 2019 to September 2023. The bars represent the number of samples collected each date, while the line indicates the prevalence (%) over the same period.

From June 2019 to September 2023, IAV circulation was detected in pigs at the Menesianos educational farm, with monthly prevalence ranging from 0% to 52.5%, as determined by a gene-specific RT-qPCR test (Figure 1). In June 2019, one of the highest prevalences observed during the study period was recorded at 25% (95% CI: 10.0 to 49.4). However, from July 2021 until February 2023, no pigs tested positive for IAV. In March 2023, positive pigs were again detected on the farm, coinciding with the return of school children and the lifting of COVID-related safety measures (Figure 1).

**Figure 2:**
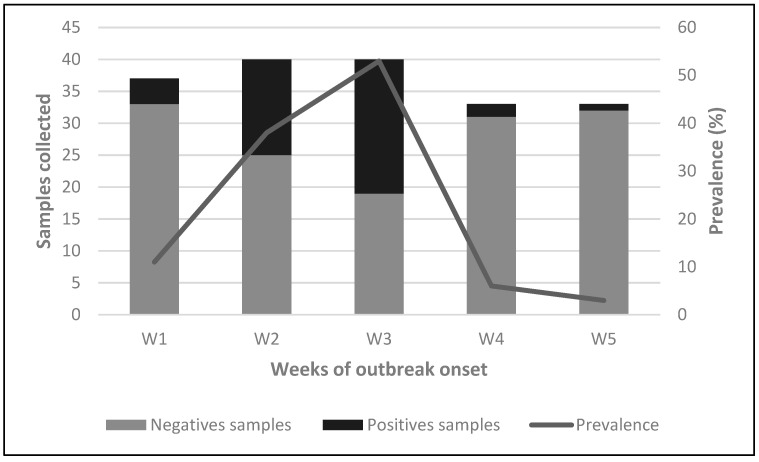
Detection of IAV in nasal swab samples during outbreak. Weekly distribution of samples collected and prevalence during the outbreak onset. Bars represent the number of negative and positive samples collected each week (W1–W5), while the line shows the prevalence (%) over the same period.

Between May and June 2023, an outbreak of IAV was detected among the swine population. The outbreak lasted five weeks with a peak prevalence during week 3 (Figure 2). At the beginning of the outbreak in week 1 (W1), no animals showed clinical signs compatible with IAV. However, four samples were RT-qPCR positive. Subsequently, in week 2 (W2), all swine on the farm underwent sampling, resulting in a prevalence rate of 37.5% (15/40). Notably, all positive animals were 4-month-old piglets housed in the same pen. Among them, only one exhibited clinical signs indicative of IAV, including low weight, general weakness, and generalized petechiae (small red spots caused by bleeding of capillaries into the skin). Regrettably, this piglet succumbed to the illness two days later and was not available for post-mortem analysis. In week 3 (W3), a third round of sampling encompassing all pigs present on the farm revealed a prevalence rate of 52.5% (21/40). The positive animals identified at this sampling point included adult breeders without clinical signs and recently weaned piglets. The piglets exhibited diarrhea at the time of sampling. In the last two weeks, the resolution of clinical signs was evident.

**Figure 3:**
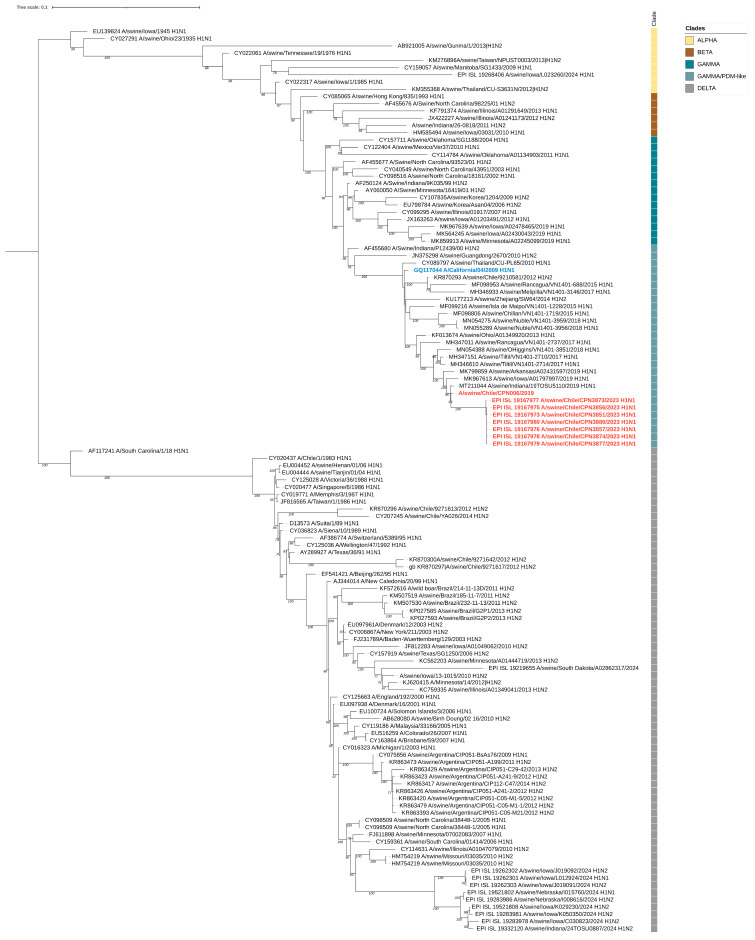
Maximum-likelihood phylogenetic analysis of the hemagglutinin gene of swine-origin influenza (H1N1) viruses from Chile sequenced for this study (red) and reference sequences. The pandemic (H1N1) 2009 influenza virus strain is highlighted in blue. Bootstrap values ≥ 70 are indicated. Major influenza virus clades are shown. Scale bars indicate nucleotide substitutions per site.

**Figure 4:**
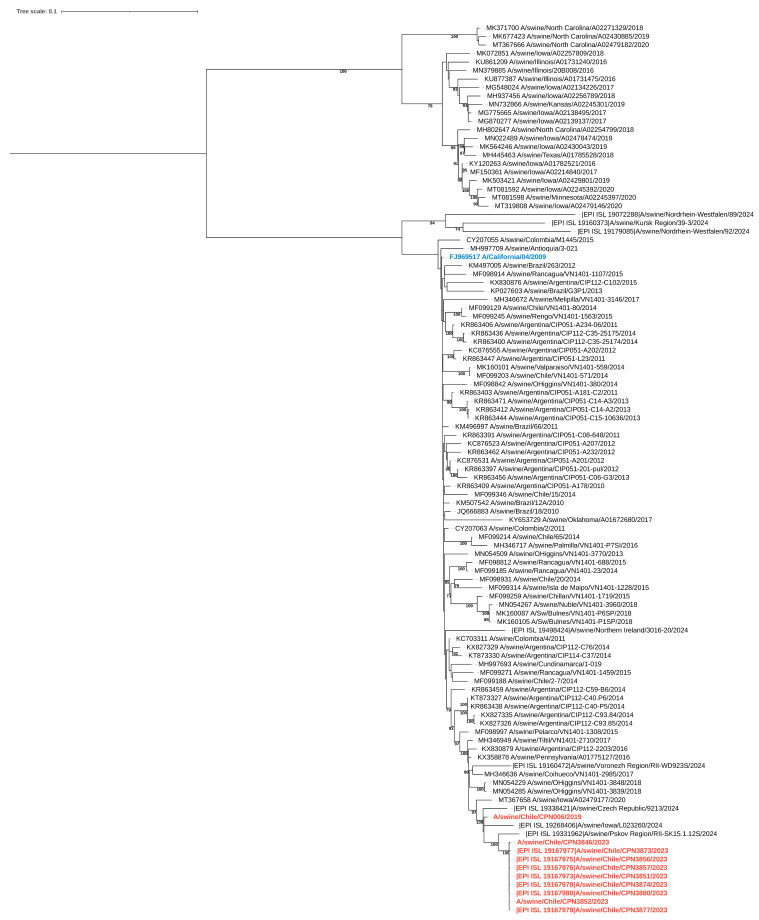
Maximum-likelihood phylogenetic analysis of the neuraminidase gene of swine-origin influenza viruses from Chile sequenced for this study (red) and reference sequences. The pandemic (H1N1) 2009 influenza virus strain is highlighted in blue. Bootstrap values ≥ 70 are indicated. Scale bars indicate nucleotide substitutions per site.

During the study, nine influenza virus sequences were obtained, including six complete and three partial genomes. Eight of these sequences were collected during the outbreak, while one was obtained in 2019. Phylogenetic analysis indicated that all genomes, regardless of the year of isolation, were related to the pandemic (H1N1) 2009 influenza virus strain (Figure 3, Figure 4 and Figure S1). The sequences exhibited nucleotide identity ranging from 99.5% to 100% and amino acid similarity between 99.3% and 100% across all genes when compared to locally circulating human strains from 2019 and 2022 (Supplementary Table S2). Notably, the sequences obtained in 2023 showed 100% nucleotide identity among themselves.

By clade assignment and mutation identifier using the FluServer platform, the 2019 sequence belonged to the 6B.1A.5a clade, whereas all other sequences obtained in 2023 were classified as 6B.1A.5a.2.1. We found that the 2019 HA sequence displayed the V240D and E206Q mutations, both associated with increased affinity for human sialic acid receptors [35,36]. On the other hand, all the 2023 sequences displayed the K173R and R240Q mutations, both also associated with increased affinity for alpha2,6-linked sialic acid receptors [37,38].

## 4. Discussion

This study is the first in South America to document the circulation of the influenza virus on an educational farm, emphasizing the critical need for IAV surveillance at this interface. The close contact between animals and humans, particularly children and young adults, presents a significant risk for pathogen transmission.

Several studies in the United States have shown that exhibition swine represent a critical interface for bidirectional transmission of influenza virus between swine and humans, with sporadic outbreaks of H3N2v influenza reported in people attending agricultural fairs [14,17,23,39]. Educational farms, which feature pigs alongside other domestic animals on exhibit, closely resemble the systems found at agricultural fairs. However, to date, no studies have investigated the transmission of IAV in these settings.

Since 2019, our research group has been actively conducting surveillance for IAV on various educational farms. This effort has provided valuable insights into IAV circulation among pigs and led to the detection of an outbreak at one of these farms. The observed outbreak lasted 5 weeks (ranging from the week of the 22nd of May to the week of the 19th of June 2023); however, only a few pigs exhibited an influenza-like illness. The pigs that showed clinical signs were young pigs (4 months old), one of which died during the second week. This is consistent with the literature, as influenza A infection in pigs typically lasts for 6–7 days. Clinical signs, including fever, respiratory distress, and weakness—sometimes accompanied by diarrhea—generally resolve within a few days [40]. Infection is usually mild and rarely causes death [2]. However, it is important to note that even in a very small population (with only 40 pigs), viral circulation can last up to 5 weeks and affect up to 90% of pigs.

Phylogenetic analysis of the sequenced viruses indicated that all genomes were related to the pandemic (H1N1) 2009 influenza virus strain. Due to the high nucleotide and amino acid similarity between the isolated strains in humans and the closest circulating IAV strains in humans at the time of sampling, along with the fact that some farm workers reported having an influenza-like illness (ILI) during the outbreak, a possible case of reverse zoonosis could be indicated. While the described mutations—V240D and E206Q in the 2019 sequence and K173R and R240Q in the 2023 sequences—have been previously linked to increased binding affinity for human-like (α2,6-linked) sialic acid receptors, their presence in our sequences should be interpreted with caution. These findings, while noteworthy, do not necessarily indicate a functional advantage and may simply reflect natural genetic variation or sequencing artifacts. Nonetheless, they highlight the importance of continued molecular surveillance to detect potential adaptive trends.

After the initial spread of the H1N1pdm in the human population, multiple countries documented that the H1N1pdm virus was circulating in pigs [18,41,42,43,44]. Much of the international spread of swine IAVs has been attributed to the movement of infected humans introducing the viruses to pigs as well as the movement of live pigs in trade [19]. In the case of this educational farm, no new animals were introduced into the herd during the weeks before the outbreak. Therefore, it is most probable that the virus was transmitted to the pigs by an infected person, and then the swine-to-swine transmission within the herd would have been responsible for additional animal infections. This situation has already been documented in other similar outbreaks around the world [18]. However, because no diagnostic test was performed on the farm workers, this hypothesis could not be verified.

It is important to highlight that both the farm workers and the students who practice with the pigs do not usually wear personal protective equipment during the handling of the animals, such as gloves and masks. This poses a risk of bidirectional transmission of IAVs both by aerosols and fomites [18]. Respiratory droplets produced by an infected pig through coughing and sneezing can remain suspended in the air, giving a nearby pig or human the chance to inhale those droplets and the resulting viral particles, potentially leading to infection [23]. In addition, fomites in the swine barn may constitute another route of indirect transmission of IAV in the educational farm environment.

Studies conducted at swine fairs in the United States have demonstrated the importance of contaminated surfaces in the transmission of IAVs [23,24]. Environmental samples were collected from feeders, waterers, and chute surfaces during corralling activities. IAV was detected via RT-qPCR in 18.0% of environmental samples. In addition, genotypes of the recovered IAV isolates were associated with variants of influenza infections in humans [24]. Therefore, environmental contamination at sites where pigs are on exhibition may also represent a risk for transmission to people visiting these sites. These findings reinforce the need for new and improved mitigation strategies, such as rinsing with water, cleaning, and wiping down environmental surfaces with disinfectants, to reduce the risk to animal and public health [23,24].

All of the previously discussed evidence demonstrates that educational farms represent a significant risk for the recombination of viruses circulating between people and pigs, particularly during the winter months (May–August), which coincide with the influenza virus season in the human population in the southern hemisphere [45]. Furthermore, the absence of positive cases from July 2021 to February 2023, followed by the reappearance of IAV in March 2023, coincided with the return of school children and the lifting of COVID-related safety measures, underscoring the potential role of human–swine interactions in viral transmission dynamics.

## 5. Conclusions

Knowledge about disease risks at educational farms remains limited. Our research demonstrates that zoonotic and potentially reverse zoonotic events can occur in these settings, underscoring the critical need for ongoing disease surveillance. This is especially important given that students, aged 8 to 17, are exposed to potentially infectious sources during visits to these farms.

## Data Availability

The data presented in this study are available on request from the corresponding author.

## Supplementary Materials

The following supporting information can be downloaded at: https://www.mdpi.com/article/10.3390/v17050635/s1, Figure S1: Maximum-likelihood phylogenetic analyses of internal genes of swine-like influenza viruses from Chiles, Table S1: List of NCBI and GISAID accession numbers for the influenza virus genomes generated and analyzed in this study, Table S2: NCBI and GISAID accession numbers for influenza virus genomes used in the phylogenetic analyses of the HA segment in this study, Table S3: NCBI and GISAID accession numbers for influenza virus genomes used in the phylogenetic analyses of the NA segment in this study, Table S4: NCBI and GISAID accession numbers for influenza virus genomes used in the phylogenetic analyses of the MP segment in this study, Table S5: NCBI and GISAID accession numbers for influenza virus genomes used in the phylogenetic analyses of the NP segment in this study, Table S6: NCBI and GISAID accession numbers for influenza virus genomes used in the phylogenetic analyses of the PA segment in this study, Table S7: NCBI and GISAID accession numbers for influenza virus genomes used in the phylogenetic analyses of the PB1 segment in this study, Table S8: NCBI and GISAID accession numbers for influenza virus genomes used in the phylogenetic analyses of the PB2 segment in this study, Table S9: Pairwise identity matrix comparing influenza virus sequences obtained in this study with human-derived sequences from the same time period available in GISAID, Table S10: Amino acid substitutions detected in the HA segment of influenza virus genomes obtained in this study, using FluSurver and based on comparisons with reference strains.
