# Supplementary figures and images for: Circulation and Spillover of pdmH1N1 Influenza A Virus at an Educational Swine Farm in Chile, 2019–2023
Source: Viruses. 2025 Apr 28;17(5):635. doi: 10.3390/v17050635 (PMC12115865; doi:10.3390/v17050635)

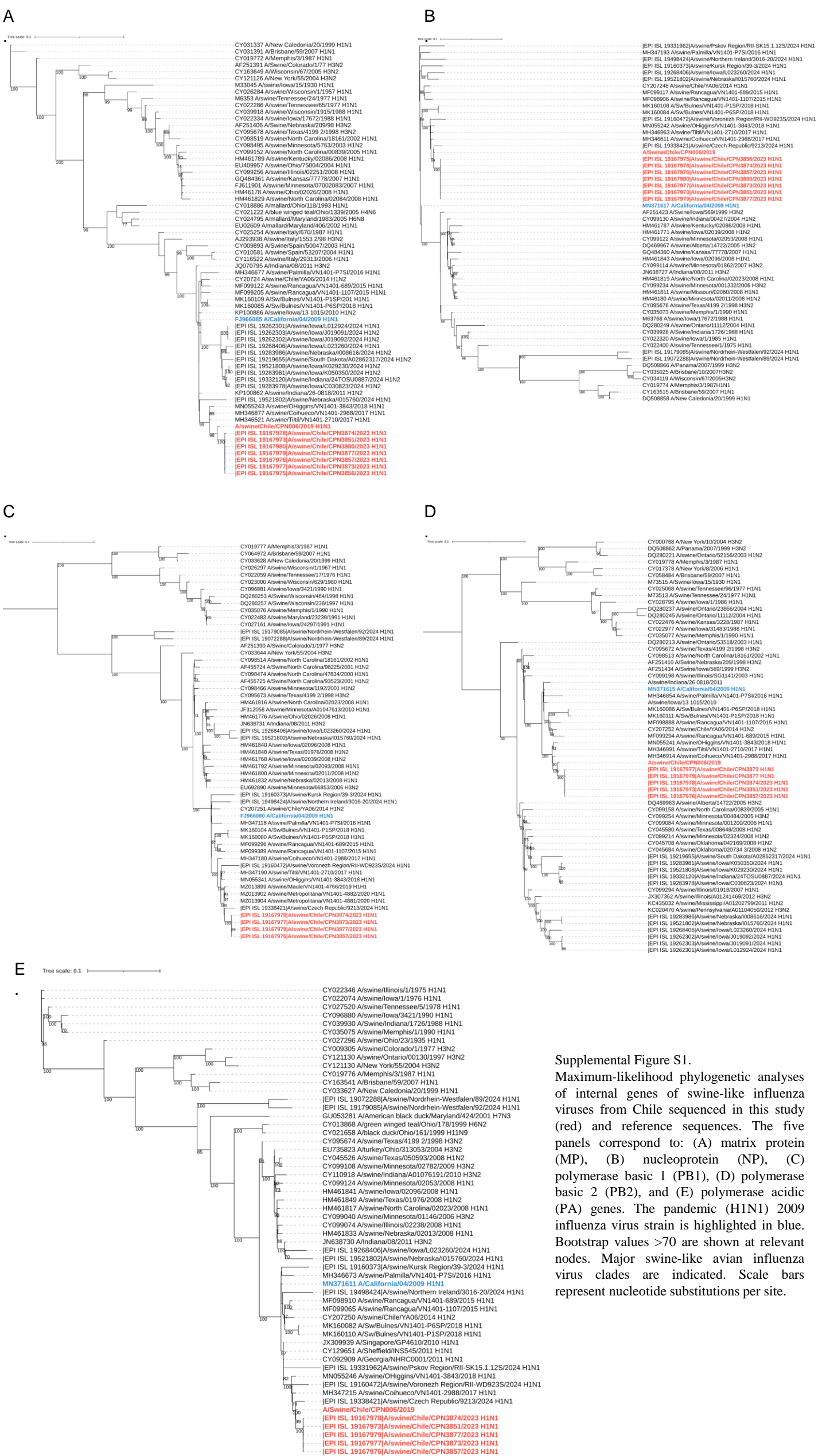

Supplement: Supplementary file 1 [file viruses-17-00635-s001.zip › Supplementar Figure 1_V2.pdf]
